# Understanding Glymphatic’s Role In Proteinopathies (GRIP): A single centre case-control study protocol

**DOI:** 10.1371/journal.pone.0360617

**Published:** 2026-10-29

**Authors:** Sofie Lysholm Lian, Tryggve Holck Storås, Ebba Müller Gløersen, Grethe Løvland, Einar Hopp, Ingrid Mossige, Markus Hovd, Hilde Thelle Uggerud, Kristin Reimers Kardel, Heidi A. Bye, Anne Lise Buset, Kirsten Valdmanis, Anne Brita Knapskog, Anne Rita Øksengård, Tormod Fladby, Kristian Bernhard Nilsen, Kent-André Mardal, Geir Ringstad, Per Kristian Eide, Mathias Toft, Kaja Nordengen

**Affiliations:** 1 Department of Neurology, Oslo University Hospital, Oslo, Norway; 2 Institute of Clinical Medicine, University of Oslo, Oslo, Norway; 3 The Intervention Centre, Oslo University Hospital, Oslo, Norway; 4 Department of Physics and Computational Radiology, Oslo University Hospital, Oslo, Norway; 5 K.G. Jebsen Centre for Brain Fluid Research, Oslo, Norway; 6 Division of Radiology and Nuclear Medicine, Oslo University Hospital, Oslo, Norway; 7 Department of Psychology, Oslo University Hospital, Oslo, Norway; 8 Department of Pharmacy, University of Oslo, Oslo, Norway; 9 Department of Transplantation medicine, Oslo University Hospital, Oslo, Norway; 10 Norwegian Climate and Environmental Research Institute (NILU), Kjeller, Norway; 11 User representative, Norwegian Health Association, Oslo, Norway; 12 User representative, Norwegian Parkinson Association, Oslo, Norway; 13 Department of Geriatric Medicine, Oslo University Hospital, Oslo, Norway; 14 Norwegian Health Association, Oslo, Norway; 15 Department of Neurology, Akershus University Hospital, Lørenskog, Norway; 16 Department of Neurophysiology, Oslo University Hospital, Oslo, Norway; 17 Department of Numerical Analysis and Scientific Computing, Simula Research Laboratory, Oslo, Norway; 18 Department of Mathematics, University of Oslo, Oslo, Norway; 19 Department of Neurosurgery, Oslo University Hospital, Oslo, Norway; PLoS ONE, UNITED STATES OF AMERICA

## Abstract

Alzheimer’s disease (AD), Parkinson’s disease (PD), and dementia with Lewy bodies (DLB) are proteinopathies characterized by pathological accumulation of amyloid beta, phosphorylated-tau, and alpha-synuclein. Impaired clearance of these proteins is thought to contribute to disease pathogenesis, whereas preserved or enhanced clearance may be compensatory. Beyond intracellular degradation within neurons and glial cells, an extracellular clearance pathway, the glymphatic system, facilitates cerebrospinal fluid flow into perivascular spaces, exchange with interstitial fluid, and subsequent clearance from the brain. Glymphatic transport may therefore be critical for removal of excess proteins and metabolic waste, and its dysfunction could promote protein aggregation and neurodegeneration. The Glymphatic’s Role In Proteinopathies (GRIP) study is an observational case-control imaging and biomarker study comparing glymphatic clearance in patients with AD, PD, and DLB versus healthy controls. We aim to recruit 70 participants: PD (n = 20), DLB (n = 10), AD (n = 20), and age- and sex-matched healthy controls (n = 20). The AD group will be stratified by pathology, with half amyloid-positive/tau-negative (A + T-) and half amyloid- and tau-positive (A + T+). The PD group will be stratified by disease duration. Participants will undergo baseline MRI followed by intrathecal injection of gadobutrol (0.25 mmol) and serial MRI at 5, 24, 48, and 72 hours. Influx and efflux of tracer will be modeled from T1 signal changes. Blood will be sampled repeatedly from 1 to 72 hours; cerebrospinal fluid will be collected at baseline. Individual glymphatic clearance will be estimated using population pharmacokinetic methods, where gadobutrol serves as a tracer. Sleep will be monitored with triaxial accelerometry and sleep diaries. The primary outcome is group differences in glymphatic clearance metrics between each disease group and controls; secondary analyses will explore associations with cognition, disease severity and duration, sleep quality, and cerebrospinal fluid protein levels.

## Introduction

Parkinson’s disease (PD), Alzheimer’s disease (AD) and dementia with Lewy bodies (DLB), also known as neurodegenerative disorders or proteinopathies, are multisystemic disorders with a huge disease burden involving cognitive decline and reduced life expectancy, in addition to motor symptoms. Currently no curative treatment exists. To develop effective therapeutic strategies, we need a deeper understanding of the underlying pathology of these diseases. Common for all three disorders is progressive neurodegeneration following progressive protein aggregation. While PD and DLB is neuropathologically characterized by aggregation of α-synuclein-rich Lewy bodies, AD is neuropathologically characterized by aggregation of amyloid-β in amyloid plaques and hyperphosphorylated tau in neurofibrillary tangles. Additionally, co-pathology is common among these diseases [1,2]. The glymphatic system is a glia-dependent clearance pathway that removes toxic waste products from the brain by cerebrospinal fluid (CSF) driven transport to interstitial space [3] and is proposed to play a central role in the pathogenesis of neurodegenerative diseases [4]. The current gold standard for measuring glymphatic clearance is to measure either how fast an exogenous CSF- tracer is cleared from the brain, or how fast an intrathecally administered CSF tracer reaches peripheral blood [5]. One way to measure glymphatic clearance rate in humans is through gMRI; injection of magnetic resonance imaging (MRI) contrast (gadobutrol) in the spinal canal followed by subsequent MRI scans to measure changes in contrast distribution within the CSF spaces, through the brain parenchyma to dural lymphatic vessels [6] and eventually to cervical lymph nodes [7]. In previous studies this has been performed in patients with idiopathic normal pressure hydrocephalus [8], subdural hematoma and subarachnoid hemorrhage [9], by quantitative measures of the amount of centrally administered gadobutrol cleared to peripheral blood [10,11] and under sleep-deprivation [12]. It has been shown that gadobutrol transport varies considerably among individuals and diseases [13,14].

Several other MRI modalities have been used to assess glymphatic function, including diffusion tensor imaging along perivascular spaces (DTI-ALPS) within the deep white matter [15–17]. However, the approach is strongly influenced by white matter microstructure and does not directly quantify cortical transport of substances [18,19]. In parallel, several non-invasive techniques are emerging. Ultra–high-field MRI, defined as field strengths of 7 T or higher in humans, provides a substantially increased signal-to-noise ratio (SNR) and enables improved spatial resolution compared with conventional systems [20]. This combined with modified T2-prepared imaging has identified region-specific drivers of CSF motion within the subarachnoid space (SAS) [21]. Heartbeat-synchronized dynamic DI has further enabled the characterization of fluid dynamics across the SAS, cortex, and white matter [22]. Despite these advances, the use of a gadolinium-based tracer in CSF remains the current gold standard for *in vivo* assessment of glymphatic function. Gadobutrol has been compared to Iodixanol, where the former showed stronger correlations with distribution in CSF [23].

The overall aim of this study is to investigate the efficiency of the extracellular waste clearance system by using intrathecal gadobutrol in patients with three different neurodegenerative disorders compared with healthy controls.

## Materials and methods

### Study cohort

The “Glymphatics Role In Proteinopathies” (GRIP) study aims to recruit a total of 70 participants, comprising patients with PD (*n* = 20), DLB (*n* = 10) and AD (*n* = 20). In addition, an age- and sex-matched control group of healthy individuals *n* = 20 will be included to facilitate comparative analyses across patient groups. Controls will be matched to the patient cohort to ensure balanced comparisons with respect to age and sex. See figure.

To be eligible for inclusion, participants must have a confirmed diagnosis of PD, DLB or AD for the patient groups, and be without a neurological disorder for the control group. Furthermore, both controls and patients must be between 50 and 75 years of age, and be able to provide written informed consent, as summarized in Fig 1. The PD cohort will be diagnosed according to the MDS criteria [24] and stratified into two subgroups: newly diagnosed PD (diagnosis within the past 2 years; *n* = 10) and established PD (diagnosis more than 5 years prior; *n* = 10). Disease duration is defined from the time of formal clinical diagnosis, including confirmation by a positive dopamine transporter (DAT) scan. The AD cohort will similarly be subdivided to capture different stages of the disease trajectory: half of the participants (*n* = 10) will have evidence of amyloidosis without hyperphosphorylated tau (A + T-) and the other half (*n* = 10) will have both amyloidosis and tauopathy (A + T+) as confirmed by CSF biomarkers [25], using the revised Alzheimer’s Association criteria [26]. Probable DLB with MCI will be diagnosed by an experienced neurologist or geriatrician according to current diagnostic criteria [27].

**Fig 1:**
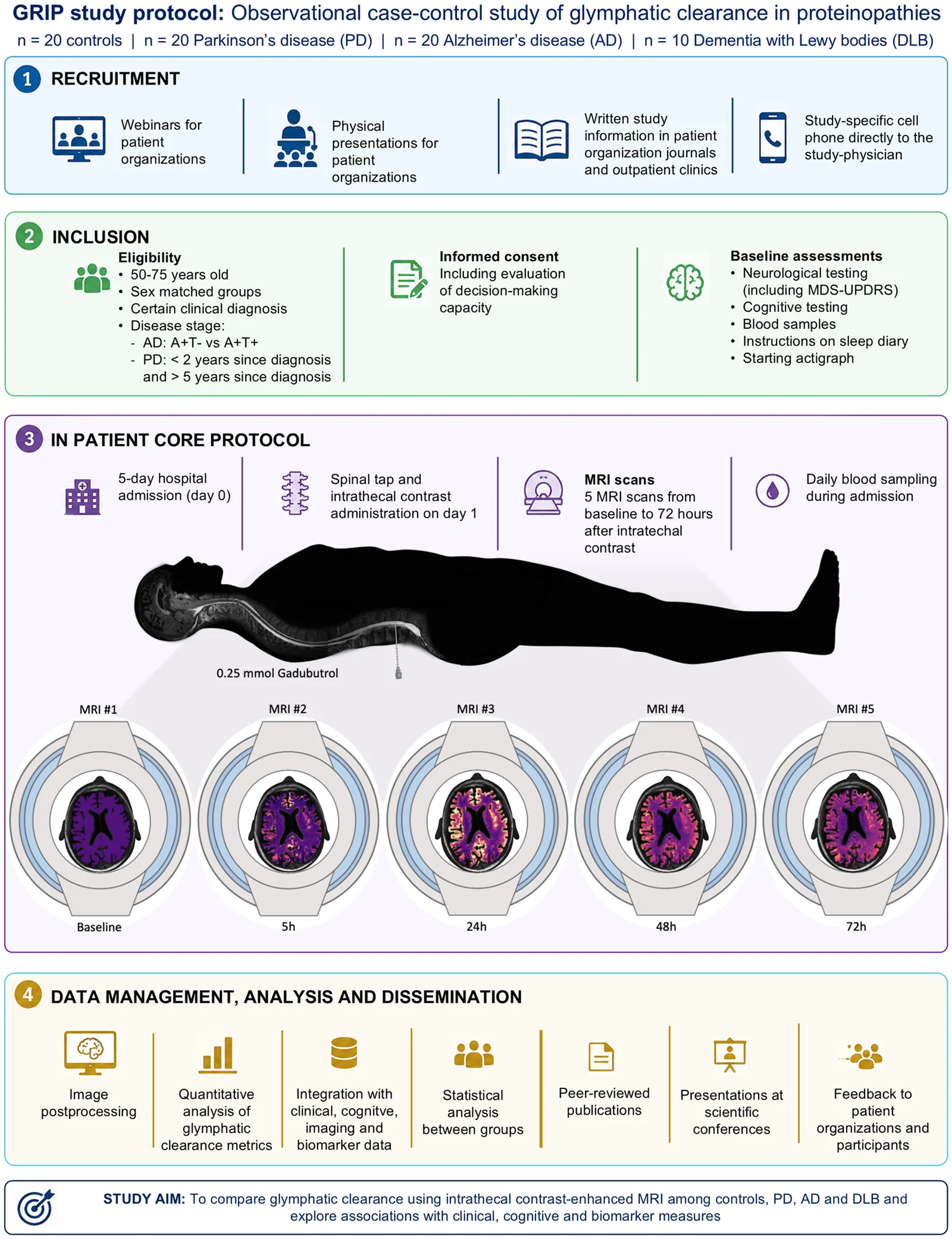
GRIP study protocol consisting of four important phases: 1) recruitment, 2) inclusion, 3) in-patient core protocol and 4) data management, analysis and dissemination. The figure is made using AI-generated icons and MRI scans projected over icons from BioRender.

Patients with PD, DLB and healthy controls will be recruited through the neurological and geriatric outpatient clinics at Oslo University Hospital, Akershus University Hospital and via the Norwegian Parkinson Association and Norwegian Health Association. Recruitment strategies include webinars, podcasts, written study information distributed through newsletters, and oral presentations at local association meetings. Patients with AD will be recruited primarily from the Memory Outpatient Clinic at the Department of Neurology at Akershus University Hospital, based on CSF profiles consistent with AD pathology, in addition to advertisements and the Norwegian Health Association.

Potential participants will receive comprehensive information about the GRIP study both orally and in written form. The ability to provide informed consent is an essential requirement for participation; individuals with cognitive decline of such severity that it affects adequate understanding of the study procedures will therefore be excluded.

Additional exclusion criteria include: any history of hypersensitivity or allergic reactions to contrast agents; reduced kidney function (low estimated glomerular filtration rate, eGFR < 60mL/min/1.73m2); a history of severe allergic reactions; ongoing treatment with antiplatelet/anticoagulant medication; thrombocytopenia; and the presence of neurological disorders other than PD, DLB, or AD.

At inclusion we will record height and weight, vital signs (blood pressure, temperature, heart rate, oxygen saturation and respiratory rate), as well as previous medical history and current medications.

### Ethical considerations

All participants in this study will be provided with comprehensive information about the study purpose, procedures, potential risks, and benefits. Informed consent will be obtained from each participant, ensuring their full understanding, voluntary participation and the possibility of withdrawing consent. The study is approved by the Regional committee for medical and health research ethics (REC) South East (#282297) and the Data protection official (DPO) at Oslo University Hospital (21/19051). Every effort will be made to minimize potential harm to participants. The participants will be carefully monitored to ensure their well-being throughout the imaging process. MRI sequences will be performed in prioritized order, to ensure the most important data points in case of the necessity of shortening down the scanning time due to discomfort. Since gadobutrol is an off-label tracer, it is important to note that the dose of 0.25 mmol was carefully selected based on safety studies [28].

The research team will continually monitor the study to identify and address any emerging ethical concerns or issues that may arise during the research. All collected data will be securely stored in compliance with data protection laws and regulations. Access to sensitive data will be restricted to authorized personnel only.

### Cognitive and neurological testing

Each participant will undergo a standard clinical examination, with addition of the Movement Disorder Society Unified Parkinson’s Disease Rating Scale (MDS-UPDRS) part I-IV [29]. Global cognitive function will be tested with Montreal Cognitive Assessment (MoCA) [30]. Learning, delayed recall and recognition will be tested using the word list memory and learning neuropsychological battery Consortium to Establish a Registry for Alzheimer’s Disease (CERAD) [31], executive function using the Trail Making test part A and B [32], verbal phonemic fluency (Controlled Oral Word Association test (COWAT/FAS)) [33]. Cognitive impairment will be determined when results are 1.5 standard deviation below the normative mean within one or more cognitive domains, and applied norms will be local, and regression based [34–38].

### Sleep assessment

All participants will be given a sleep diary with written and oral instructions for a total of two weeks (14 days), starting prior to gMRI and lasting at least a week after the gMRI protocol. Throughout this time, the participants will also be given a triaxial accelerometry (actigraphy) worn on the arm, provided by the Section of Clinical Neurophysiology. Additionally, five self-assessment sleep scales will be given REM Sleep Behavior Disorder Screening Questionnaire [RBDSQ] [39], Parkinson’s Disease Sleep Scale [PDSS-2] [40], Pittsburgh Sleep Quality Index [PSQI] [41], Insomnia Severity Index [ISI] [42] and Circadian Type Inventory [CTI] [43]. The study physician will offer instructions.

### Glymphatic MRI (gMRI)

Included patients and controls will undergo a four-day protocol with five MRI sessions. The baseline MRI protocol consisting of T1 weighted (T1w), T2 weighted (T2w), diffusion tensor imaging (DTI), fluid-attenuated inversion recovery (FLAIR), susceptibility-weighted imaging (SWI), parenchyma T1-mapping [44] and T2w CSF T1-mapping [45]. While T1w and T2w will be used for anatomical segmentations, DTI will be used to model patient-specific white matter tracts and FLAIR and SWI, together with T1w and T2w will be used diagnostically to uncover subclinical previous cerebral events or anatomical abnormalities or variations. After the baseline MRI protocol, patients will undergo a lumbar puncture in the lateral position with a G25 atraumatic needle, where proper needle placement will be confirmed through a passive release of CSF. CSF for analyses will be collected. The MRI contrast agent Gadobutrol will be administered intrathecally as a CSF tracer at a dose of 0.25 mL of 1.0 mmol/mL (Gadobutrol; Gadovist, Bayer Pharma AG, Berlin, Germany) to enable quantification of CSF clearance and visualization of glymphatic influx and CSF transport. If needed, an interventional radiologist will perform an X-ray guidance lumbar puncture in the lateral position.

Subsequent MRI scans will be performed at approximately 5 hours, 24 hours, 48 hours and 72 hours after contrast injection, and consist of the T1w and the T1-mapping sequence for quantitative estimation of contrast enhancement in brain parenchyma and a T2w T1 mapping for segmentation of intracranial CSF spaces and for quantitative contrast enhancement in CSF [45]. A 3D T1-weighted Turbo Field Echo (T1w-TFE) sequence will be used for anatomical reference.

All MRI examinations will be performed on a 3T Philips Ingenia scanner (Philips Healthcare, Best, The Netherlands) using a 32-channel head coil.

The T1 mapping sequences and the T1w-TFE will be full brain coverage 3D acquisitions with 1 mm isotropic resolution. In addition to the above sequences, the baseline protocol includes standard clinical and glymphatic MRI sequences (1 mm isotropic 3D FLAIR, 0.7 mm isotropic 3D T2w imaging, susceptibility-weighted imaging [SWI], and 2.5 mm isotropic axial DTI [b-values 0, 1000s/mm^3^]), resulting in a total baseline scan duration of approximately 60 minutes. Follow-up imaging sessions will use a subset of these sequences, with a total scan time of approximately 30 minutes per session.

### CSF handling and biomarker analysis

CSF will be collected immediately before intrathecal contrast administration. CSF will be collected in polypropylene tubes and processed according to the BIOMARKAPD protocol [46]. Briefly, samples will be centrifuged within 4 hours at 2,000 x *g* for 10 minutes at room temperature, aliquoted, and stored at −80°C until analysis. Additional aliquots will be stored for future exploratory biomarker analyses.

Routine CSF analyses will be performed immediately at the local laboratory, including cell count, lactate, glucose, total protein, and albumin ratio. A clinical validation of amyloidosis and tau pathology will be validated using commercial enzyme-linked immunosorbent assays (ELISAs) based on monoclonal antibodies. This will be used to determine CSF levels of amyloid-β 42, total tau, and phosphorylated tau (Innotest β-Amyloid (1–42), Innotest hTau Ag, and Innotest Phospho-Tau (181P); Fujirebio, Ghent, Belgium).

### Blood sampling and analysis

Serial venous blood sampling will be performed to assess gadobutrol clearance from CSF to peripheral blood. Intravenous blood will be collected before intrathecal contrast administration and at 1, 2, 4, 6, 8, and 10 hours thereafter, followed by sampling every 4–6 hours for the patients admitted to the hospital the whole period. To reduce patient burden of sampling, blood will preferentially be drawn via an intravenous cannula. Before administering contrast, we will check platelet count (>150), creatinine, estimated glomerular filtration rate (eGFR < 60mL7min/1.73m^2^, CKD-EPI2021) and signs of infection (leukocytosis or elevated C-reactive protein). Additionally, standard clinical chemistry will be performed for all the participants.

To further reduce patient burden, we will perform volumetric absorptive microsampling of capillary blood using the Mitra (Trajan Scientific and Medical) devices. This allows the patient to perform self-sampling, thus more flexible sampling of capillary blood.

At the pre-contrast time point five Vacutainer tubes will be collected (one serum tube and four EDTA tubes). At all subsequent time points, one EDTA tube will be obtained. Samples will be stored in a fridge at 4°C until processing. For biobanking, tubes will be centrifuged at 1,500 x *g* for 15 minutes, plasma and serum aliquoted into 500 μl matrix tubes and stored at −80°C until analysis. Two EDTA tubes of whole blood will be stored directly without any pre-processing. For participants who do not require continuous hospitalization during the 5-day study period, at-home sampling will be performed with dried Mitra® blood spot analysis using capillary blood. For determination of total gadolinium (Gd) associated with gadobutrol contrast agent in peripheral blood, the plasma sample will be thoroughly mixed by repeated aspiration and dispersing with a pipette. Approximately 0.3 g of plasma will be transferred to a digestion vessel and the exact mass recorded prior to microwave-assisted digestion with concentrated ultrapure nitric acid and deionized Milli-Q water (3 mL water and 5 mL nitric acid). Digestion will be performed in an UltraCLAVE single reaction chamber microwave system (Milestone) using an 80-minute stepwise heating program, with a maximum temperature of 250°C held for 20 minutes. After cooling to room temperature, digested samples will be transferred to 50 mL polyethylene vials (VWR) and diluted with deionized water, in accordance with previously published protocols [10]. Gadolinium concentrations will be determined by inductively coupled plasma mass spectrometry (ICP-MS; Agilent 7700x, single- quadrupole MS; Agilent Technologies). Indium will be added online as an internal standard to all samples, calibration standards, blank samples and quality control samples to correct for variations in sample introduction, instrumental drift and matrix effects. NIST SRM 1515 Apple Leaves or NIST SRM 1547 Peach Leaves will be used as certified reference materials (CRMs) to provide an independent quality control of the sample digestion and ICP-MS determination of gadolinium, and to support the assessment of the trueness of gadolinium determination through comparison with the available indicative values.

### PET imaging

[^18^F]Flutemetamol PET will be performed in *n* = 40 of the participants (*n* = 20 AD patients, *n* = 10 DLB patients and *n* = 10 controls). There are no specific preparations before [^18^F]Flutemetamol PET. All subjects will be given a bolus injection of approximately 185 MBq (5 mCi) intravenously. Subjects will be positioned head-first supine in the scanner followed by a low-dose CT scan acquisition for attenuation correction and anatomical co-localization. PET scanning in 3D-mode will start approximately 90 min after injection of [^18^F]Flutemetamol. PET data will be acquired in list-mode for 20 min (four frames of five minutes) and image reconstruction will be performed according to guidelines [47]. [^18^F]Flutemetamol PET images will be corrected for motion, if necessary, by removing afflicted frames obtained in list-mode. Attenuation corrected images co-registered with low-dose CT will be visually assessed for global and regional cortical tracer uptake according to validated standards by two experienced nuclear medicine physicians [48].

Semi-quantification will be performed in PMOD with co-registration of PET images to 3D T1 MRI. For global amyloid burden the Centiloid atlas will be applied. Image processing will be performed in batch analysis with inspection of VOI placement as quality control. Standard uptake value ratio (SUVR) with normalization to whole cerebellum will be converted to Centiloids (CL) and PMOD processing will be performed according to previously validated pipeline [49].

For regional amyloid burden we will measure [^18^F]Flutemetamol SUV in segmented cortical regions from the FreeSurfer pipeline. Whole cerebellum will be used as reference region to obtain regional SUVRs.

### Image analysis and postprocessing

MRI images will be converted from Philips Enhanced DICOM files to NIfTI format using the dcm2niix software tool (https://github.com/rordenlab/dcm2niix) and data will be organized in a standardized structure similar to Brain Image Data Structure [50].

Brain segmentation and surface reconstruction will be performed using FreeSurfer’s recon-all processing pipeline [51–53], or similar automated segmentation tools [54], based on the pre-contrast 3D T1-weighted image. For automated CSF segmentation, our newly developed tool will be used (open-source on GitHub; https://github.com/jorgenriseth/grip-mixed.git). The CSF segmentation is created by thresholding a pre-contrast heavily T2 weighted image, and extrapolating brain segmentation labels to the CSF mask for SAS segmentation. All segmentations will be manually assessed by a neuroanatomist.

To enable tracking the regional time evolution of the tracer distribution, the contrast-enhanced MRI time series (5 hours, 24 hours, 48 hours and 72 hours) will be registered to the pre-contrast T1-weighted image for each participant. Greedy, FreeSurfer, or similar tools, will be used for image registration. T1 relaxation rates scales with tracer concentration. Hence, T1 maps from the T1-mapping sequences will be calculated to quantitatively assess the intrathecal tracer distribution in the brain and CSF. Furthermore, normalization of T1-weighted signal intensities enables semi-quantitative analysis of tracer distribution.

Diffusion tensor images will be corrected for susceptibility-induced [55] and eddy current-induced (FSL’s eddy) distortions before calculating the diffusion tensor and diffusion maps (FSL’s dtifit) [56]. An additional b = 0 scan with opposite phase encoding direction (anterior-to-posterior) to the DTI acquisition will be used as input for susceptibility correction.

### Quantitative analysis of glymphatic clearance

To measure regional gMRI, we will use automated segmentation of the subarachnoid space that corresponds to specific regions of the cerebral cortex. This tool allows us to analyze gadolinium availability in these regions accurately. By integrating and analyzing both PET data and regional gMRI metrics enable us to focus on specific sub-regions of the cerebral cortex, facilitating a thorough investigation of the relationship between glymphatic clearance and protein aggregation.

Population pharmacokinetics methods for individual quantification of glymphatic clearance have previously been demonstrated to be of clinical significance, and associates with biomarkers of neurodegeneration [11]. By employing a rich sample regimen, especially in the initial absorption phase of intrathecally administered gadobutrol, we will be able to obtain accurate and precise estimates of individual glymphatic clearance.

These imaging-derived measures will then be integrated with clinical, cognitive, neuroimaging, and biomarker data. The primary outcome will be group differences in glymphatic clearance quantified from gMRI data and comparing each disease group with healthy controls.

Secondary analyses will examine associations between glymphatic clearance and cognitive performance, disease severity, disease duration, and CSF and PET protein levels.

### The type of data and statistical analyses planned

Descriptive statistics will be calculated for all variables, and group differences between PD, AD, DLB, and controls will be assessed with appropriate parametric or non-parametric tests, with an initial emphasis on transparent characterization of the raw data.

To explore physiological mechanisms related to CSF circulation, glymphatic function, and clearance pathways, we will perform multi-physics modeling ranging from single one-compartment diffusion and advection simulations to complex multi-compartment fluid-structure interaction problems involving poro-elasticity, describing the transport of water, contrast and proteins between CSF, brain parenchyma, blood and dura. These models range from currently computationally feasible on a cohort of 70 [57,58] to complex multi-physics models that are currently not feasible [59,60] with the present methods. We do however anticipate significant improvement in the computational methodology in the following years.

These models will provide parameters such as transfer and clearance rates and effective distribution volumes, which will be compared between diagnostic groups and controls to identify potential disease-related alterations.

The analysis will proceed in phases. The first phase will focus on FreeSurfer-derived structural and CSF-related measures in PD patients versus healthy controls. In the second phase, the study will be expanded to include 20 AD and 10 DLB patients, with a shared or disease-specific control group, to determine whether CSF- and contrast-related patterns in PD generalize to or differ from those in AD and DLB. Across all phases, we will relate these imaging findings to clinical parameters, including cognitive function, sleep patterns, and other relevant measures, to compare profiles between the diagnostic groups.

Group differences in glymphatic clearance will be assessed using parametric tests (e.g., t‑tests or analysis of variance [ANOVA]) for normally distributed data, and non‑parametric tests (e.g., Mann-Whitney U test or Kruskal-Wallis test) for non‑normally distributed data. Associations between glymphatic clearance and cognitive performance, disease severity, disease duration, and CSF/PET protein levels will be examined using linear regression models for normally distributed variables and Spearman rank correlations for non‑normally distributed or ordinal variables. Model assumptions (normality, homoscedasticity, and absence of multicollinearity) will be checked using standard diagnostic procedures, and data transformations or robust/non‑parametric methods will be applied if assumptions are violated. Analyses will be adjusted for relevant covariates (e.g., age, sex, education, and intracranial volume), and multiple comparison correction will be applied where appropriate (e.g., false discovery rate).

### User involvement

The GRIP study’s design, including participant information and consent procedures, has been developed in close collaboration with user representatives. These representatives have provided valuable input to ensure that patient perspectives are adequately addressed and that the consent materials are clear, comprehensible, and appropriately balanced regarding risks and benefits. An example of this is the length of MRI scanning. Another important feedback point from the user representative is the need for regular updates to study participants, as the direct patient involvement is intensive and brief, while the overall project duration is expected to be long. We therefore plan to provide updates to participants one to two times per year, either through seminars or informational letters. User representatives will be involved in discussions about the implications of the results, the need for further research, and suitable formats for communication to their members.

### Status and timeline

Recruitment for this study began in summer 2022 and is ongoing, and so far, the PD patients and half of the controls are included. Inclusion is planned to continue to 2031. No formal data analyses on case-control differences glymphatic clearance have been performed to date.

## Discussion

The GRIP study has several important methodological strengths. Unlike previous work, where gMRI has primarily been performed on clinical indication, this is the first study to apply gMRI purely for research purposes. The GRIP study will also perform gMRI in age-matched healthy controls, providing a more appropriate defined comparison group. Technically, this project advances beyond earlier gMRI studies that relied on conventional T1-weighted MRI with normalization to small regions of reference tissue (e.g., retroorbital fat) for semi-quantitative assessment of contrast enhancement [13]. These approaches are vulnerable to variability within small normalization regions. GRIP will instead exploit the fixed gray scale of the Look-Locker sequence to obtain quantitative measures in the brain parenchyma without dependence on such normalization, thereby reducing susceptibility to regional reference-tissue variability. Furthermore, to overcome limitations of using Look-Locker for CSF measurements, the project has developed a new MRI sequence that selectively detects the CSF signal [45]. This allows sensitive detection of subtle baseline signal differences reflecting variations in protein concentration, as well as quantitative R1 measurements in contrast-enhanced CSF. Basing the work on T1 mapping represents an advancement over previous approaches. More recently, this framework has been further refined as T1 mapping itself has technical challenges [61]. In addition, amyloid PET will be performed in AD and DLB patients, enabling comparison of regional protein aggregation with regional glymphatic clearance.

Another strength of the protocol is the extended follow-up time. Previous studies typically followed patients for up to 48 hours, with an additional scan after 4 weeks after intrathecal contrast administration and showed peak contrast enhancement at around 24 hours in most brain regions [10] with continued increase between 24 and 48 hours in deep white matter and deep nuclei such as the basal ganglia. To ensure that the phase of maximal efflux is captured, the GRIP study extends imaging to 72 hours.

The GRIP study also integrates comprehensive fluid biomarker assessment. Whereas earlier gMRI studies often administered intrathecal contrast without CSF sampling, GRIP includes CSF collection for biomarker analyses, including diagnostic markers. Compared with prior work, the study incorporates more frequent peripheral blood sampling, particularly immediately before and after sleep, to better characterize potential differences in clearance profiles between sleep and wakefulness. Finally, all participants will undergo out-of-lab sleep monitoring and will complete sleep diaries, allowing sleep quality, sleep fragmentation and sleep duration to be related directly to measures of glymphatic clearance.

The main limitation in the study design is the time-consuming MRI scanning protocol and potentially tiresome investigations. Furthermore, both lumbar- and venipuncture for CSF sampling, intrathecal injection and blood sampling might be bothersome for the study participants. For lumbar puncture including intrathecal MRI contrast administration there is a considerable risk of post-puncture headache some days following the procedure. Appropriate measures will be taken to reduce the risk (thin needle (G25) and atraumatic needle technique) and follow-up of the participant after the procedure with necessary care [62]. We acknowledge that the protocol is likely to be distressful for some participants, and we will follow each closely to ensure that all possible discomfort will be as low as possible.

The study requires frequent blood sampling during the first day of admission at the hospital ward. Blood will primarily be drawn through an indwelling venous catheter (cannula) to reduce discomfort and the number of separate needle insertions. However, additional blood draws by standard venipuncture may be necessary if cannulas clog. To further minimize discomfort and reduce the volume of venous blood required, we will also employ volumetric absorptive microsampling of capillary blood. This technique allows measurement of gadobutrol concentrations from small capillary samples, thereby limiting the need for repeated venous punctures.

This interdisciplinary study needs coordinated effort across multiple units at both the hospital and the university, making close collaboration particularly critical on the days when patients are admitted for inclusion. The PhD fellow will have a leading role in this process, ensuring that all involved units are appropriately informed and adequately prepared in advance of each inclusion.

### Dissemination plan

The results from this project will be disseminated through several complementary channels targeting both the scientific community and relevant stakeholders, including patients, patient organizations, health services, and the public. We will submit the main findings to international, peer-reviewed journals, and separate manuscripts are planned to cover the MRI processing methods and quantitative calculations of the difference between glymphatic clearance in the three neurodegenerative disorders compared to healthy controls.

The project results will also be presented at relevant national and international scientific conferences. Study participants will receive feedback in the form of a short lay summary of the overall study results once the main analyses are completed. Clinically relevant individual findings, such as incidental MRI findings, will be communicated according to predefined clinical routines and in line with ethical approvals. All information for participants will be provided in clear, non-technical language, and participants will be offered the opportunity to ask questions.

Key findings will be communicated to relevant patient organizations through written summaries in lay language and, when appropriate, through short presentations at meetings, podcast or webinars.

## Abbreviations

AD: Alzheimer’s disease
PD: Parkinson’s disease
DLB: Dementia with Lewy bodies
CSF: Cerebrospinal fluid
SAS: Subarachnoid fluid/space?
MRI: Magnetic resonance imaging
gMRI: Glymphatic MRI
GRIP: Glymphatic’s Role In Proteinopathies
DaT: Dopamine Transporter
MOCA: Montreal Cognitive Assessment
CERAD: Consortium to Establish a Registry for Alzheimer’s Disease
COWAT: Controlled Oral Word Association test
VOSP: Visual Object and Space Perception
TMT: Trail Making Test
ELISA: enzyme-linked immunosorbent assay
EDTA: Ethylenediaminetetraacetic acid
T1w: T1 weighted
T2w: T2 weighted
DTI: Diffusion tensor imaging
FLAIR: Fluid-attenuated inversion recovery
Mixed: Mixed spin-echo inversion recovery
PET: Positron Emission Tomography
REM: Rapid Eye Movements
RBDSQ: REM Sleep Behavior Disorder Screening Questionnaire
PDSS-2: Parkinson’s Disease Sleep Scale
PSQI: Pittsburgh Sleep Quality Index
CTI: Circadian Type Inventory
ISI: Insomnia sleep index
SCD: Subjective Cognitive Decline
MCI: Mild Cognitive Impairment
CSF: Cerebrospinal fluid
MDS-UPDRS: Movement Disorder Society Unified Parkinson’s Disease Rating Scale
